# Experimental study on high temperature tensile behaviour of aluminium alloy AA5083 with oscillating load

**DOI:** 10.1038/s41598-023-40527-5

**Published:** 2023-08-16

**Authors:** Mohammad Shirinzadeh Dastgiri, Zackary Fuerth, Leo Kiawi, Daniel Green

**Affiliations:** Department of Mechanical, Automotive & Materials Engineering, University of Windsor, Windsor, ON N9B 1K3 Canada

**Keywords:** Mechanical engineering, Mechanical properties, Metals and alloys

## Abstract

The flow behaviour of aluminium alloy AA5083 at 450 $^\circ$C has been investigated under quasi-static loading conditions with and without a superimposed oscillating load. Samples were placed under tensile load at constant strain rates ranging from 0.001 to 0.3 s$^{-1}$. A fixture was designed to generate the required sine-wave oscillation and was attached to the MTS tensile test machine along with a secondary, highly sensitive load cell. The frequencies of the imposed oscillations ranged from 5 to 100 Hz with an amplitude ranging from 0.02 to 0.5-N. It was observed that the imposition of oscillations influences the deformation behaviour of the material. Although the yield and tensile strength remain relatively constant, the total elongation is 8–23% higher under an imposed oscillating load. In addition, the thickness distribution profiles along the gauge length of the tensile specimens were investigated and it was observed that in the presence of oscillations the thickness distribution is more uniform. It was concluded that the presence of a superimposed oscillative load will result in greater deformation capabilities before fracture and postpone the occurrence of damage compared to conventional forming. This phenomenon was further explored utilising a user-defined material subroutine developed for the finite element solver LS-DYNA to simulate the conducted constant load tensile tests.

## Introduction

Aluminium AA5083 is a non-heat-treatable aluminium alloy with excellent cold formability and can reach moderate levels of superplasticity^1^. This alloy is affordable and has reasonable environmental resistance and good mechanical properties, making it ideal for aerospace, marine and automotive applications^2^.

### Introduction to superplastic forming

With the high demand and development of parts and products in the automotive and aeronautic industries, the need for lightweight parts and improved forming processes has increased drastically. Therefore, a lot of research is involved in improving lightweight aluminium alloys such as AA5083 and its different forming processes. A material is said to exhibit superplastic behaviour when it exhibits significant plastic deformation (an elongation $> 200\%$) at elevated temperature without necking prior to fracture^3^. Both the mechanical properties (elongation, UTS, optimal forming temperatures, strain rate dependence, etc.) and the microstructural characteristics of superplastic aluminium alloys have been extensively investigated^4–10^. There are three main aspects necessary for the achievement of superplasticity: high forming temperatures (approximately half of the melting point of the material), fine and stable grain size (less than 10 $\upmu$m), and a low and controlled rate of deformation, typically between 10$^{-4}$ and 10$^{-2}$ s$^{-1}$. A lot of research has gone into the optimisation of these aspects, Hosseinipour ran tests to obtain optimal temperatures and strain rates, concluding that 450 $^\circ$C and strain rates of magnitude 10$^{-3}$ s$^{-1}$ achieved optimal results^11^. Yogesha et al.^12^ reported similar combinations of high temperature and low strain rate requirements in order to deform superplastically.

Of particular interest, published research has shown the effectiveness and results of superplastically deforming AA5083 sheets^11,13^. The severe deformations occurring in superplastic forming are primarily achieved by grain boundary sliding (GBS). Furthermore, high levels of grain boundary sliding are accompanied by an additional accommodating deformation mechanism, and traditional GBS models are separated into two categories: diffusionally accommodated and dislocationally accommodated GBS  ^14^. Despite the very large deformations that can be achieved, the main drawback to the widespread use of superplastic forming is the significant time required to form an industrial part, ranging from two to ten minutes^15^. This limits the number of parts that can be formed, especially in the automotive industry, which significantly increases the cost of the part.

Blow-forming processes can take advantage of superplastic materials to plastically form complex parts having a relatively uniform thickness distribution. In this process, a metallic blank may be preheated and then placed in a die that is heated to the prescribed forming temperature. A pressure vs. time curve is used to control the pressure of the hot sheet against the surface of the die cavity; with air or argon gas, forcing the blank into its final shape  ^16^. This process has successfully reduced the weight of several parts used in the automotive and aerospace industries.

### Improvement of superplastic capabilities and processes

If superplasticity could be achieved at strain rates faster than those of typically slow superplastic-forming strain rates, industrial forming processes could be enhanced. High Strain Rate (HSR) superplasticity has been the target of much research to boost superplastic forming rates. As a result of the correlation between increased maximum elongation and reduction of grain size, HSR research is primarily concerned with the refinement of grains in the microstructure. In order to refine grains and theoretically demonstrate superplasticity at high strain rates, it is the examination of severe plastic deformation processes. Although efficient, this procedure can be costly and has limited industrial application. Yan et al.^17^ performed uniaxial tensile tests on AA5083 specimens that were thermo-mechanically processed to achieve a grain size $< 8 \,\,\upmu$m (compared to commercially available AA5083 that typically has a grain size $> 10 \,\,\upmu$m) and they achieved a maximum elongation of 530%. Ma et al.^18^ achieved elongation greater than 1000% using an aluminum alloy that had been processed by friction stirring to achieve a grain size $< 2 \,\,\upmu$m. Jin et al.  ^19^ utilised grain refinement through friction stir welding to achieve superplasticity at elevated strain rates up to 10$^{-2}$ s$^{-1}$.

The effectiveness of superposition of an oscillating load to optimise both metal plastic deformation and several metal forming processes has been investigated for decades, as seen in ultrasonic welding^20,21^. Ultrasonic welding uses high-frequency vibrations to weld two clamped components, and the result is a highly repeatable and high-strength weld. Moreover, superimposing ultrasonic oscillations in tensile tests to improve deformation has also been investigated^22–27^. Superimposed oscillations hypothetically improve the effectiveness of deformation due to the oscillatory stress generating a mean stress lower than that of the stress strain curve for a static tensile load.

The effectiveness of imposing ultrasonic oscillations onto a metal that is plastically deformed can be related to the effectiveness of heat as it pertains to thermal softening. However, the results in the literature indicate increased effectiveness compared to thermal softening because energy is preferentially absorbed in localised areas which typically impede deformation; vacancies, dislocations, and grain boundaries, while the entire volume of the material absorbs the thermal energy. Due to its effectiveness in optimising deformation capabilities, research on the application of oscillation energy during manufacturing has been under development. Langenecker et al.^27^ investigated the use of ultrasonic energy during metal forming, resulting in a reduction of the force to iron and deep-draw copper shells from 220 to 70 lb. In other metal forming processes, the success of inducing ultrasonic oscillations was attributed to the oscillations reducing the friction between the part and the forming tools. Reducing frictional effects in superplastic forming processes is of great value to reduce post-forming costs and processes to improve post-formed surface finishes, which are traditionally negatively impacted by sliding during forming. The consensus for oscillations/vibrations ability to aid in deformation is energy being preferentially absorbed at dislocation sites, allowing them to then overcome slip obstacles. This is especially valuable for a part that deforms superplastically to aid in dislocation-accommodated grain boundary sliding. In addition, ultrasonic vibration has been shown to aid in refining the microstructure of the material and, in turn, improving the properties of the material. The effect of introducing ultrasonic vibrations as a means of grain refinement was investigated in an ultrasonic assisted upsetting process and then compared to a conventional upsetting process^25^. This process produced results similar to those of severe plastic deformation grain refinement processes and could also be an underlying mechanism occurring during metal forming. Parallel research has been conducted into the design and utilisation of a supersonic fluidic oscillator capable to be used under superplastic conditions^28^. In conjunction, the prospective tensile behaviour improvement along with a fluidic oscillator embedded in a superplastic die can greatly expand the incorporation of superplastically formed parts because of the forming time and prospective forming temperature reduction. The effectiveness of an oscillative pressure forming process has been recently investigated by Anaraki et al resulting in an expanded forming domain when implementing an oscillative pressure forming procedure ^29^.

### Constitutive modelling of superplastic forming processes

Due to the use of variable pressure over time in industrial SPF processes, it is of great value to develop constitutive models of the flow stress that can be used to simulate the superplastic forming process. Thus, the objective of this research is to carry out experimental testing, and to use the data obtained to develop a constitutive model of superplastic deformation with and without oscillations.

Superplastic deformation is regarded as a creep process that lies within region II of the sigmodal stress vs. strain rate curve. This region is often defined by a strain rate sensitivity index ($m={\Delta (\log {\sigma })}/{\Delta (\log {\dot{\varepsilon }})}$) that exceeds 0.4. Additionally, the activation energy in Region II is close to that required for grain boundary diffusion to occur. As a result, maximum ductility occurs within Region II, so it is often characterised as the Superplastic region. The importance of the strain rate sensitivity index is further seen with its use in all the most cited viscoplastic constitutive equations. These constitutive models include the power law, sinh law, the Bird-Mukherjee-Dorn equation, and the unified constitutive model^30^. Despite previous models using a constant m value, recent studies have indicated that this is inaccurate and can negatively skew the predictions. Additionally, despite the power law being frequently utilised in FE simulations due to its simplicity, it has limitations due to its inability to predict either the softening or the damage behaviours which typically occur during superplastic forming.

This study aims to improve the current framework by developing a constitutive model that takes into account the softening and damage behaviour of the material, as well as the constant change in the material’s strain rate sensitivity. In addition to that, the simulation of an oscillation-enhanced superplastically forming material is also developed.

## Experimental procedure

### Specimen preparation

The samples were made from 1.4 mm thick AA5083 sheets, the chemical composition of which is shown in Table 1 as provided by the material supplier. Sub-size specimens were cut to a specified geometry adopted from the literature^11^, consisting of an 18-mm gauge length and an 8-mm gauge width. As recommended in previous studies^31^ specimens were prepared using wire EDM so that the major stress axis was always parallel to the rolling direction of the sheet. This sub-sized specimen geometry is advantageous because it ensures that the specimen will fracture before the gauge length stretches outside the limits of the available furnace. This specimen geometry also facilitated the comparison of the results with data published by other researchers.

**Table 1:** Chemical composition of AA5083 (%).

Alloy	Fe	Mg	Mn	Cu	Si	Cr	Ti	Zn	Others	Al
AA5083	0.20	4.75	0.8	0.04	0.15	0.15	0.05	0.05	$<0.15$	Balance

### Testing equipment

**Figure 1:**
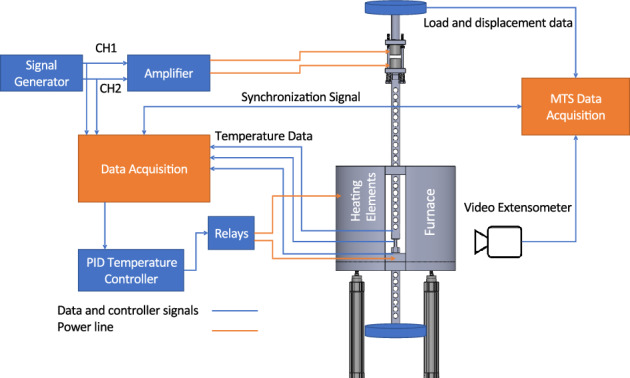
Schematic of the experimental setup.

The custom-built equipment used to carry out the experiments includes a well-insulated furnace with a double-layer quartz window, a pneumatic opening mechanism, titanium rods and fixture to hold the specimen, an electromagnetic mechanical waveform generator, an electric waveform generator, a National Instruments data acquisition and control device, and a secondary load cell. Tests were conducted on an MTS tensile testing machine. Figure 1 shows a schematic of the test setup.

**Figure 2:**
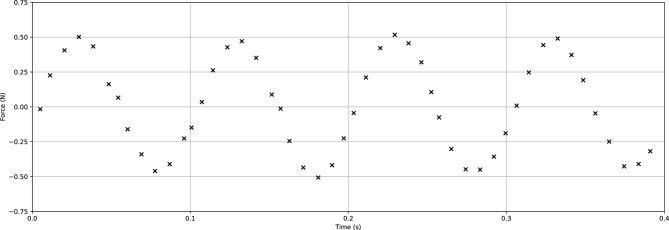
Recorded oscillation wave for a 0.5 N amplitude and 10 Hz frequency oscillating wave.

The furnace contains electric heaters that surround its two semicircular chambers for a uniform temperature distribution according to the ASTM E2448-18 standard for determining the superplastic properties of aluminium. The pneumatic lifter is used to raise and lower the furnace when mounting the specimen. Two steel rods enter through the openings in the top and bottom of the furnace to hold the specimen, as to well as transfer the load from the MTS testing machine. The openings in the furnace are thermally sealed with silica cotton during operation. One of the rods is attached to a pair of electromagnets that induce oscillations in the loading direction. Sine wave signals are generated by a waveform generator, and the electromagnets convert the signals into an oscillating load that is mechanically transferred to the specimen through the steel rods. The applied oscillating load was calibrated for each strain rate and frequency using the MTS load cell. Figure 2 shows a recorded load oscillation wave at 0.5 N amplitude of 10 Hz frequency with compensated preload.

### Testing procedure

Tensile tests were conducted in accordance with the ASTM E2448-18 standard, except for the geometry of the sample. For each condition, 3 repeat tests were performed. Before testing, the gauge length and width of each sample were measured using Mitutoyo calipers for precision. Once the oscillating load was calibrated, the furnace was closed and preheated to 450 $^\circ$C. And once the temperature reached 450 $^\circ$C, the furnace was raised and the specimen was mounted on the gripping fixtures. Temperature probes were placed in contact with the top and bottom of the specimen and held in place with steel wire. The furnace was then lowered and sealed. The start time and temperature were recorded. Once the furnace temperature stabilised at 450 $^\circ$C, the sample was left to soak for five minutes, according to industrial practice. During this process, the crosshead position was adjusted to minimise the stress build-up due to thermal expansion. Then the oscillating load was activated and the tensile test was started. For constant strain rate testing a constant monotonic loading was applied with a prescribed crosshead velocity. After the test was completed, the sample was removed and the chamber was preheated for the next test. An MTS Advantage Video Extensometer (AVX) was used to record and verify displacement data in the gauge length.

### Set-up verification

**Figure 3:**
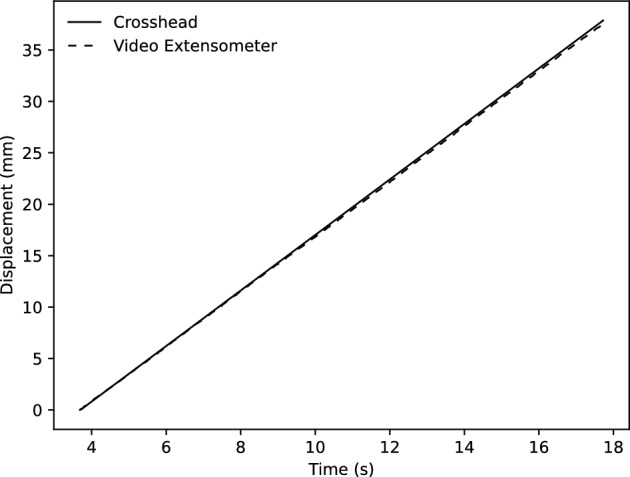
Comparison of cross-head data recorded with simultaneous readings of an MTS Advantage Video Extensometer (AVX) for a test at 0.1 s$^{-1}$ strain rate.

AVX was used to verify the accuracy of the collected crosshead data. As shown in Fig. 3. The validity of the crosshead displacement is confirmed, as shown by its strong correlation with the recorded AVX data. These data were used to confirm the elastic modulus of AA5083 at 450 $^\circ$C, and by comparing the data recorded by AVX and the crosshead data, a relation was established between the measured crosshead displacement and the gauge length displacement. This equation was used to process the load-displacement data and calculate the true effective plastic stress for each test.

## Results

**Figure 4:**
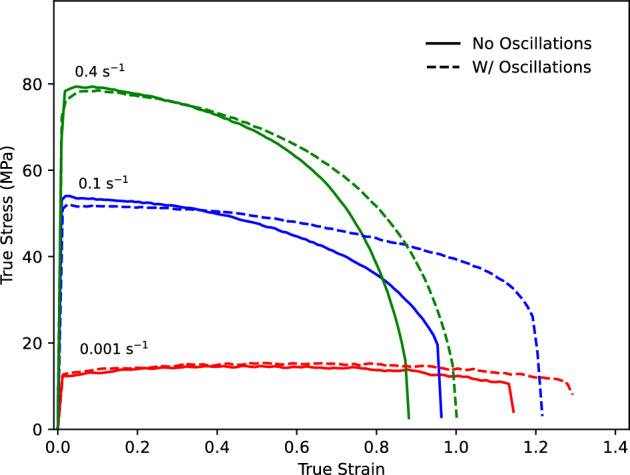
True stress strain curves from constant rate tensile tests with and without oscillations.

For material characterisation, a series of tensile tests was performed for both constant strain rate and constant force loading conditions. Using constant-rate tests to fracture, the true strain-to-fracture could be measured and compared with a model of the material’s stress-strain behaviour. After developing a material model using the results of the tensile tests, the verification of the model was carried out using the constant force tensile tests, comparing the predicted results with the results found experimentally. A compilation of the flow curves collected for the select strain rates from the tensile tests performed is shown in Fig. 4.

### Strain to fracture investigation

**Figure 5:**
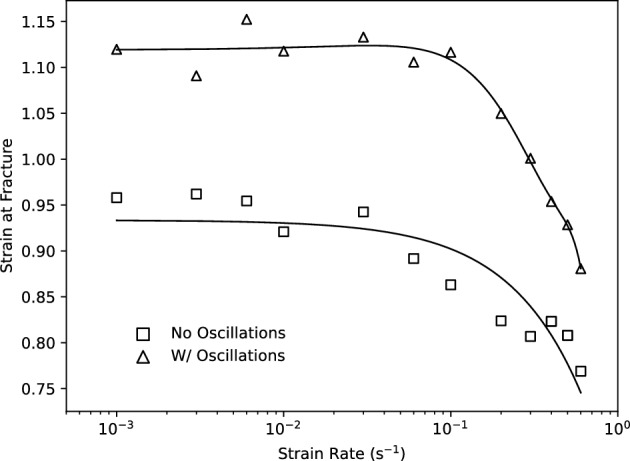
Plastic strain recorded at fracture for tensile tests conducted at strain rates ranging from 0.001 to 0.6 s$^{-1}$ with and without oscillation.

To verify the hypothesis that increased deformation occurs when an induced oscillating load is applied, tensile tests were performed at constant rates until fracture. To determine the effectiveness of the oscillating load, the displacement of the cross head at the onset of fracture was used to quantify the plastic deformation capacities at strain rates ranging from 0.001 to 0.6 s$^{-1}$. The effectiveness of the oscillating load is shown in Fig. 5, where the percentage improvements in plastic strain at fracture ranges from 13.4 to 29.4% in the strain rate domain.

### Stress-strain response with and without oscillating load

**Figure 6:**
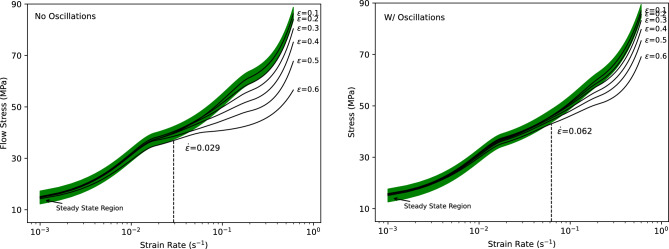
No oscillations and w/ oscillations flow stress for strain rates ranging from 0.1 to 0.6 in strain increments of 0.1.

In addition to improved formability, the stress response at different strains and strain rates also allows expansion of the applicable superplasticity domain. The domain in which superplastic forming can be applied is represented by a stable stress-strain response with increasing deformation during forming. Stability can be quantified by the limited presence of significant strain hardening or softening. The resulting relationship with and without oscillations is shown in Fig. 6 for the strain rate range of 0.1–0.6 in strain increments of 0.1 At elevated strain rates, there is a significant amount of strain softening that occurs, particularly without an oscillating load. In contrast, at lower strain rates, there is an insignificant hardness variation with increasing strain values. Compared to the behaviour with and without oscillation, the overall stress values show a strong similarity, with the main difference being a reduced overall spread, particularly at strain rates $> 0.05\,\, \hbox {s}^{-1}$ as indicated by its deviations from the prescribed steady-state region and the large deviation increases with strain values. Overall, the presence of oscillations allows for a more extensive forming region where the strain rate can be increased up to 0.06 s$^{-1}$ where it is limited to 0.03 s$^{-1}$ without oscillations.

### Impact of frequency and amplitude

**Figure 7:**
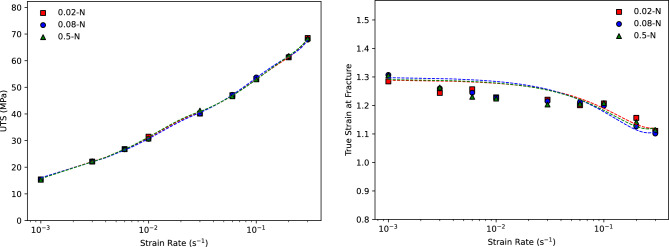
Effect of superimposed oscillation amplitude on the stress and strain behaviour of the AA5083 during tensile testing at 450 $^\circ$C.

For the purpose of conducting a thorough analysis, a wide range of frequency and amplitude combinations were investigated in relation to the stress-strain behaviour of the AA5083. Indications in literature suggest an acoustic softening effect as a result of an increase in the amplitude of the oscillating load. This is in contrast to the study conducted as illustrated in Fig. 6, there is no discernible softening effect present as a result of an imposed amplitude. In this sense, even though tests for amplitudes ranging from 0.02 to 0.5-N were performed, the magnitude of the amplitude was found to not impact the effectiveness of enhancing the formability for both stress and strain behaviour as shown in Fig.  7: no discernible difference is seen in both the maximum stress and total true strain to fracture for all of the amplitudes and strain rates applied. Similarly, the frequency of the superimposed oscillation was found to cause no variation in the deformation capabilities of the material for the range of 5–100 Hz.

### Constant loading tensile tests

**Figure 8:**
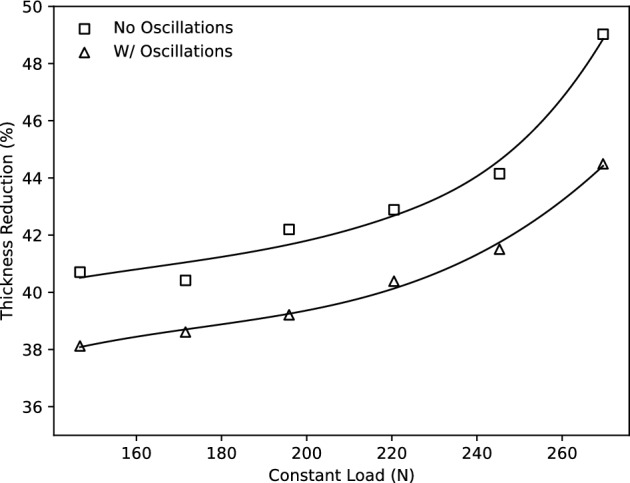
Maximum thickness reduction occurring throughout the gauge length of AA5083 specimens deformed under constant load up to 171% major strain.

To further quantify the effect of a superimposed oscillating load, as well as to validate the constructed material model, tensile tests with an applied constant load were performed to specified strain values. From the tests carried out, the thickness variation and time were recorded for comparison and material characterisation. The tests were conducted both without oscillation and with oscillation according to the selected amplitude and frequency specifications. The applied load magnitudes were within the range of 147.1–269.7-N. The relationship for the thickness reduction that occurs as a result of various applied constant loads is shown in Fig. 8. Samples were loaded to a prescribed major strain of 171 %, with and without an oscillating load, and the corresponding thickness was measured using ultrasonic thickness measurements. The resulting relationship indicates an improved resistance to thinning under equivalent loading conditions as a result of the imposed oscillations during deformation.

### Microstructural analysis

Two modes of deformation have been observed among the strain rates of the completed tensile tests: grain boundary sliding (GBS) and solute drag creep (SDC). For high temperature material deformation, GBS is more prevalent at slower strain rates but at higher strain rates, the major deformation mechanism shifts to SDC^32–34^. This is what is reported during Quick Plastic Forming, a variant of superplastic forming that is used in the transportation industry where superplastic materials are formed at much faster rates and sometimes at lower temperatures to meet the growing demands for parts.

As seen in the literature^32,34^ GBS failure is accompanied by void nucleation, void growth, and void coalescence, which then leads to fracture. In SDC, failure occurs due to flow localisation and necking in the material. It has been observed by Mary-Anne et al.^34^ that for low impurity Al-Mg materials that have undergone SDC, failure can occur by necking up till a sharp point, but for high impurity aluminum like AA5083, necking occurs under SDC deformation, however, failure still occurs by cavity growth and coalescence in the necked area.

**Figure 9:**
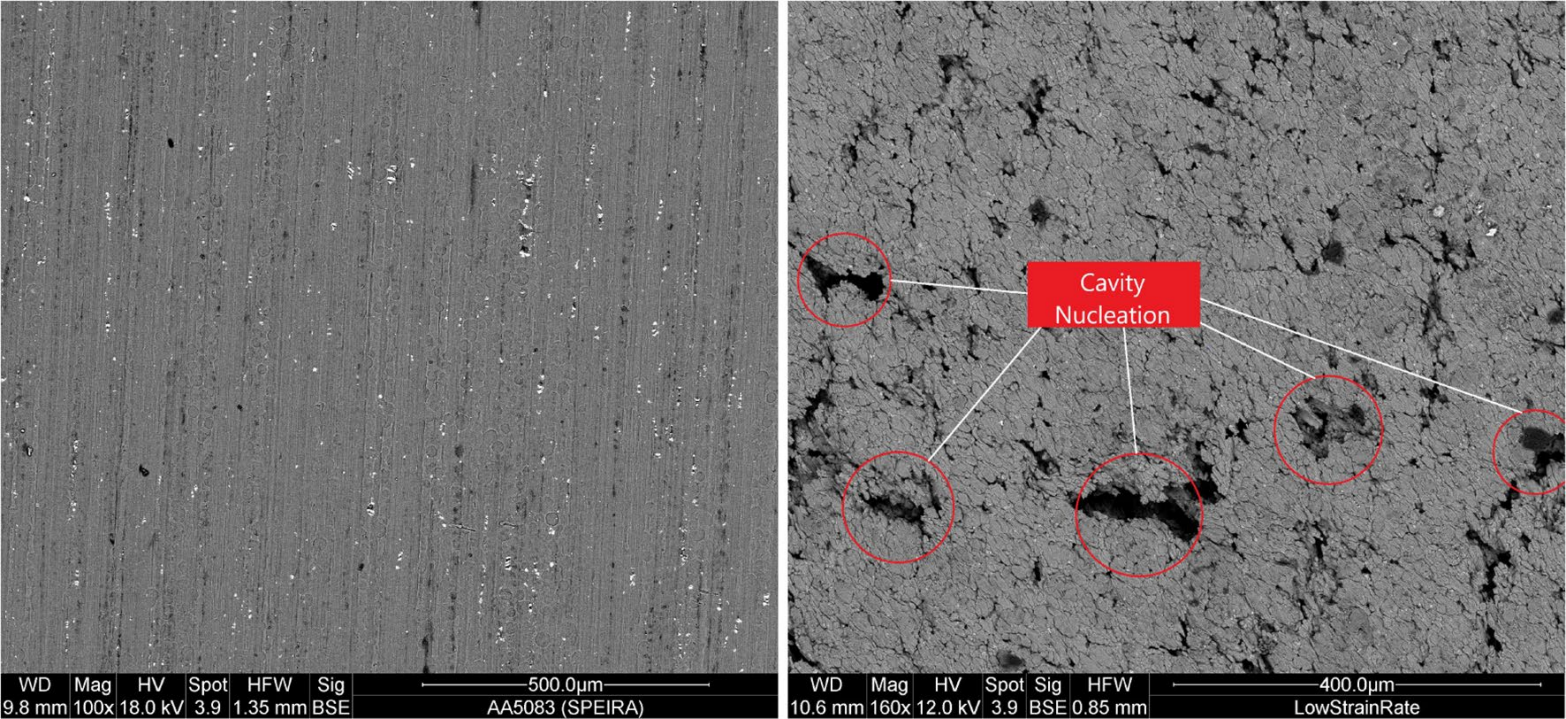
SEM images showing both an as received (left) and post formed (right) AA5083 sample showing the cavities formed in uniaxial tension at 450 $^\circ$C and 3 $\times$ 10$^{-3}$ s$^{-1}$ strain rate (tensile axis is vertical).

**Figure 10:**
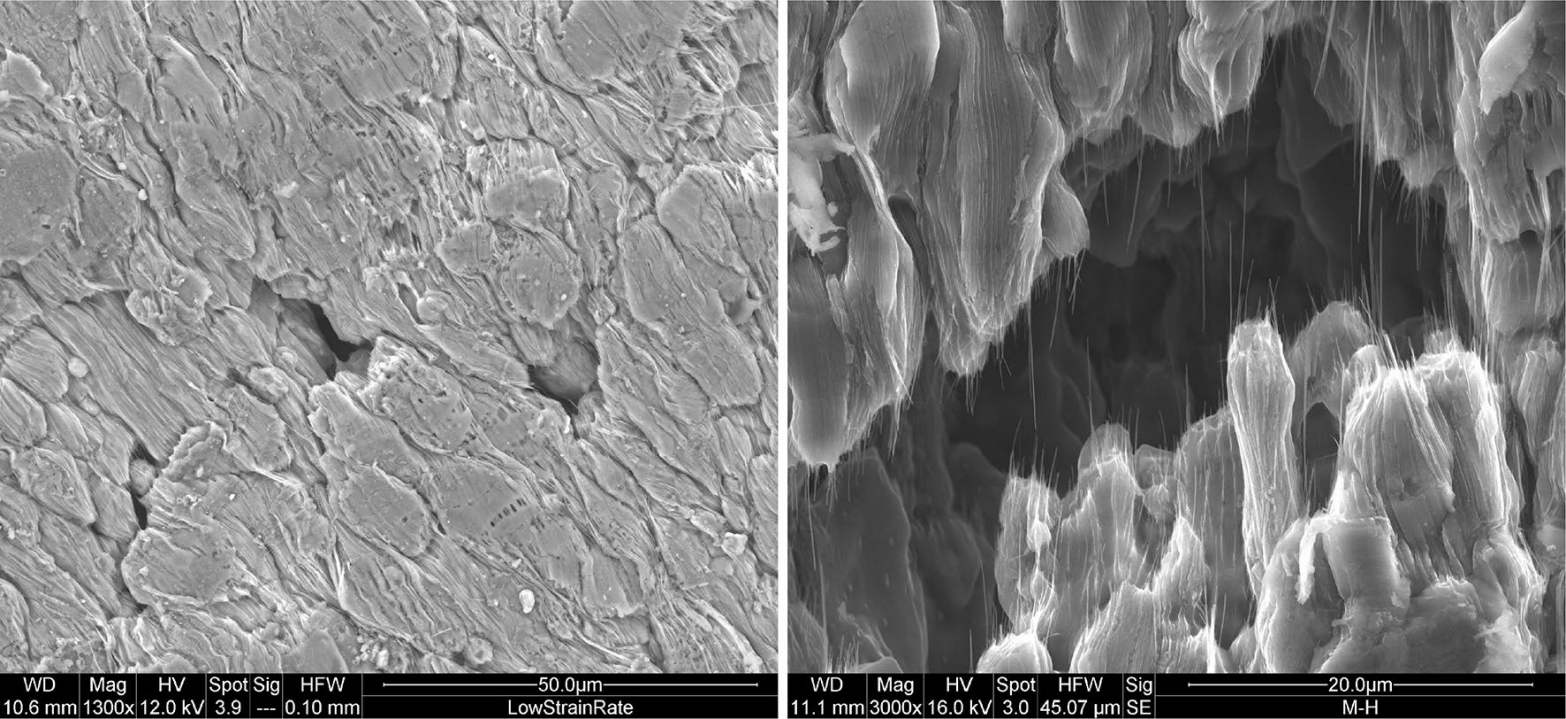
SEM images of post formed AA5083 samples deformed at 450 $^\circ$C and 10$^{-3}$ s$^{-1}$ strain rate.

SEM images of the specimens were taken before and after SPF and show deformation in the grains, cavitation development, and GBS. The image in Fig. 9 illustrates the cavities generated when deformed at 450 $^\circ$C at a strain rate of 3 $\times$ 10$^{-3}$ s$^{-1}$. The specimen shown in Fig. 10 was tested at 450 $^\circ$C with an initial strain rate of 1 $\times$ 10$^{-3}$ s$^{-1}$ and with a superimposed oscillating load. The SEM image on the left clearly reveals evidence of GBS, the grains are elongated, as expected from such high-strain deformations, and the grain boundaries have been stretched along the tensile axis (the tensile axis is at an angle of 45$^\circ$ from the vertical). In the SEM image on the right, the formation of aluminum and magnesium oxides can be seen at the grain boundaries; these oxides are characterized by a fibrous structure. This has been observed by other researchers^35,36^ who indicate that fibre formation at grain boundaries occurs during GBS at elevated temperatures. Gali et al.^36^ also obtained an Energy Dispersive X-ray (EDS) spectrum in conjunction with SEM analysis on the fibres which confirmed that they consist mainly of Al$_2$O$_3$, Mg$_2$Al$_4$O and MgO.

## Constitutive modelling

### Material model development

As previously mentioned, the power law ($\sigma = K\dot{\varepsilon }^m$) is commonly used to model superplastic deformation and forming processes. Due to the stability, characterized by a lack of significant hardening or softening in superplastic applications, the power law function is relatively accurate in most superplastic applications. However, as illustrated in Fig. 6, initially a small amount of strain hardening occurs, followed by, significant strain softening, as elevated strain rates are reached. To effectively model both mechanisms that occur, the equation outlined in Eq. (1) uses a quadratic relationship between stress and strain, along with a power law and a summation to account for the occurring strain rate hardening, where A, B, C, D, m are material parameters. Using Eq. (1), the behaviour of the material can be modelled more accurately over the entire strain rate domain (0.001–0.6 s$^{-1}$).

1 $$\begin{aligned} \sigma = \left( A\varepsilon ^2+B\varepsilon +C \right) \cdot \dot{\varepsilon }+D\dot{\varepsilon }^m \end{aligned}$$

### Determining material parameters

The outlined material parameters were then optimised separately for both conditions using the experimental results obtained from the constant-rate tensile tests with and without oscillations. The optimised material parameters for both loading conditions are presented in Table 2.

**Table 2:** Optimised material parameters for oscillating and non-oscillating loading.

	A	B	C	D	m
No Oscillations	− 261.5	85.61	18.27	88.86	0.2758
W/ Oscillations	− 210.3	88.98	12.87	84.11	0.2446

**Figure 11:**
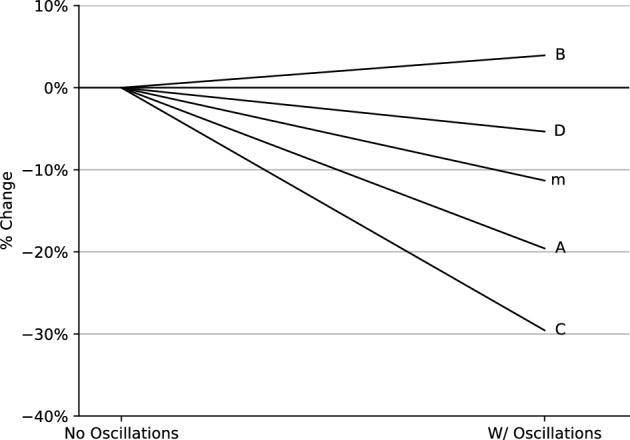
The percentage variation of the material parameters when modelling with and without oscillations.

The computed parameters were also used to describe the differing behaviour of the material with and without oscillations. The effect of the oscillations on material parameters is illustrated in Fig. 11. As shown in this figure, most of the constants decrease in value as a result of the use of superimposed oscillations. The decrease in the values defining the quadratic equation is indicative of a reduced strain softening effect as a result of oscillations. In contrast, there is very little variation for the values of D and m, indicating a lesser impact on the sensitivity to strain rate.

### Investigating quality of fit

**Figure 12:**
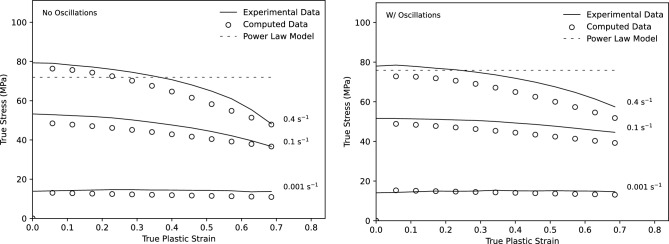
Comparison of the effectiveness of the proposed material model with a traditional fitted power law compared to the results found experimentally for strain rates of 0.001, 0.1, and 0.4 s$^{-1}$.

As previously discussed, the primary benefit of the selected model is it’s applicability over a large domain of strain rates. To illustrate the effectiveness of the constructed model in relation to the power law, three strain rates were plotted compared to the experimental data with and without oscillations according to Fig. 12. As illustrated in the figure, as strain rates exceed approximately 10$^{-1}$, the proposed model is much more effective at modelling the deformation behaviour of AA5083 at the elevated temperature.

**Figure 13:**
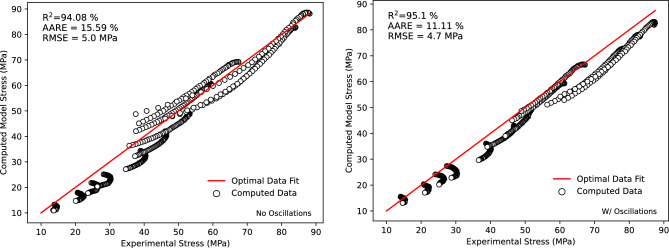
Correlation analysis of the constructed model compared to the experimental data with and without oscillations including the calculated values of $R^2,AARE$ and *RMSE*.

**Figure 14:**
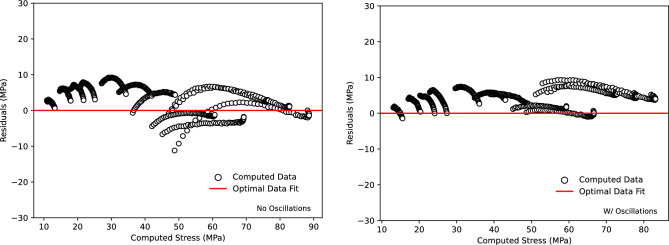
Stress residuals for computed model stress to experimental tensile test flow stress.

In addition to the qualitative and comparative analysis of the fit, a correlation analysis was conducted to evaluate the fit to the experimental data with and without oscillations. The visualisation of the quantitative correlation analysis is shown in Fig. 13. The figures show the values of $R^2,AARE$ (Average Absolute Relative Error) and *RMSE* (Root Mean Squared Error) for the computed model. The calculated $R^2$ values of 95.1 and 94.08 % for with and without oscillations, respectively, are indicative of a strong agreement between the model and the experimental results. The accompanying *AARE* and *RMSE* of 11.11 % and 4.7 and 15.59 % and 5.0 with and without oscillations, respectively, are also indicative of a strong correlation between the experimental data and the data computed using the proposed model in Fig. 1. The residuals, indicative of the variation between the model and actual stress, are shown in Fig.  14, indicating an accurate model with residuals remaining approximately within ± 10 MPa for all strain rates.

**Figure 15:**
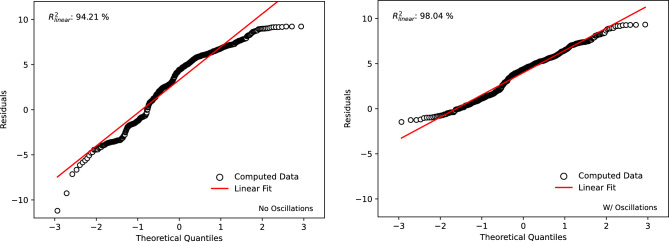
Probability plot to show the normality of residuals using correlation to a linear line of best fit.

Since the magnitude of the residuals as well as the overall correlation of the material model have been evaluated and deemed suitable, further investigation was conducted to evaluate the normality of the residuals to confirm a fully appropriate fit. To assess the normal distribution of the error terms, the probability plot of the residuals generated is shown in Fig.  15. Overall, both lines show a suitable correlation to a linear relationship with correlation coefficients of 98. 04 and 94. 21 % for with and without oscillations, respectively. The overall improvement in normality of errors and overall correlations within the oscillation model is a result of the increased stability of the stress response even at lower and elevated strain rates.

### Model verification with constant loading tests

**Figure 16:**
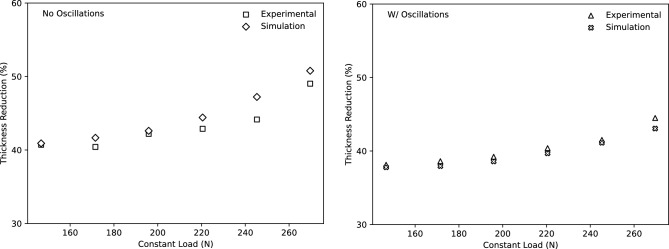
Comparison of the maximum thickness reduction in the specimen gauge measured from experiments and predicted by simulations with oscillations and without oscillations at 171% major strain.

For further numerical investigation on the process of superplastic deformation, the commercial finite element software LS-DYNA was employed. The aforementioned material model was added to LS-DYNA as a user-defined material model. Belytschko-Tsay shell elements consisting of five integration points through the thickness were used to model the tensile specimen. Rather than applying limitations as boundary conditions, the deformation was applied by allowing contact between the tensile specimen and the solid components of the test fixture. This approach takes into account the movement of the material from the gripping region to the gauge region that occurred during the conducted experimental work. The stress vs effective plastic strain in uniaxial tension was provided as input to LS-DYNA. Due to the specimen design in this instance, the experimental flow curves were not obtained in uniaxial tension, so a series of repeated simulations were run to scale the input flow curve until the experimental force displacement data could be reliably predicted. The constant load conditions shown in Fig. 8 were expected in the second series of simulations. The top fixture was continuously loaded, while the lower fixture remained fixed. The maximum thickness reduction predicted in the gauge was exported and compared to the experimental data after the simulation was set to end when it reached the 171% major strain. Figure 16 presents this comparison and shows good accuracy under all load conditions.

## Conclusions

The following conclusions can be drawn from this study.

1. The formability of AA5083 at a temperature of 450 $^\circ$C was significantly improved throughout the strain rate domain of 0.001–0.6 s$^{-1}$ using a superimposed oscillating load
2. The addition of oscillations with amplitudes and frequencies ranging from 0.02 to 0.5-N and 5 to 100 Hz respectively allowed for a relative improvement for the true strain at fracture of more than 30%
3. The stability of the flow stress and expansion of the steady state region as a function of strain rate were improved as a result of imposed oscillations during tensile deformation
4. Tensile test specimens showed a significantly lower thickness reduction when an oscillating load was superimposed
5. The experimental hardening and softening behaviour was accurately modelled using a quadratic strain relationship and power strain rate constitutive model for tensile data obtained both with and without oscillations.
6. The constructed model showed a strong correlation for the constant rate tensile tests as indicated by $R^2$ correlation coefficients of 95.1% and 94.1% for with and without oscillations, respectively.
7. Using LS-DYNA’s user-defined material subroutine, the simulation results showed a strong correlation with the constant load test data done experimentally in terms of the thickness distribution.
8. Finally, the addition of a minor oscillation during superplastic deformation leads to significantly lower rates of thickness reduction compared to conventional processes without oscillations.

## Data Availability

All data generated during this study is available by contacting the corresponding author(s) with reasonable request.
